# Effect of Nutrition Education During Pregnancy on Iron–Folic Acid Supplementation Compliance and Anemia in Low- and Middle-Income Countries: A Systematic Review and Meta-analysis

**DOI:** 10.1093/nutrit/nuae170

**Published:** 2024-11-14

**Authors:** Melaku Tadege Engidaw, Patricia Lee, Gelana Fekadu, Prasenjit Mondal, Faruk Ahmed

**Affiliations:** Public Health, School of Medicine and Dentistry, Griffith University, Gold Coast, Queensland 4222, Australia; Department of Public Health (Human Nutrition), College of Health Sciences, Debre Tabor University, Debre Tabor 6300, Ethiopia; Public Health, School of Medicine and Dentistry, Griffith University, Gold Coast, Queensland 4222, Australia; Department of Medical Research, China Medical University Hospital, Taichung City 404, Taiwan; School of Nursing and Midwifery, Griffith University, Gold Coast, Queensland 4222, Australia; School of Nursing and Midwifery, College of Health and Medical Sciences, Haramaya University, Harar 3200, Ethiopia; Public Health, School of Medicine and Dentistry, Griffith University, Gold Coast, Queensland 4222, Australia; Public Health, School of Medicine and Dentistry, Griffith University, Gold Coast, Queensland 4222, Australia

**Keywords:** nutrition education, iron–folic acid supplementation, hemoglobin, anemia, pregnant women

## Abstract

**Context:**

Stakeholders implement nutrition education to prevent and control anemia during pregnancy. Nutrition education during pregnancy can increase the consumption of iron–folic acid (IFA) supplements and encourage behavioral changes. However, there is no comprehensive meta-analysis to determine the effectiveness of this intervention.

**Objective:**

This review aimed to determine the effect of nutrition education on IFA supplementation (IFAS) compliance, hemoglobin level change, and prevalence of anemia in low- and middle-income countries.

**Data Sources:**

The systematic searches on Ovid Medline, Scopus, Embase (Elsevier), Web of Science, Health and Medical Collection (ProQuest), and Google Scholar were conducted until September 11, 2023. The updated searches were performed on November 16, 2023.

**Data Extraction:**

In total, 12 436 records were imported to Covidence. Of these, 9109 records were screened by title and abstract. A total of 112 records were evaluated in full, and 53 articles were ultimately included based on eligibility.

**Results:**

Fifty-three peer-reviewed research articles met the inclusion criteria, involving 13 475 pregnant women. Those who received nutrition education during pregnancy were 2.80 times more likely to comply with IFAS (odds ratio = 2.80; 95% CI: 2.04, 3.83; I^2^ = 66.20%). There was an average increase of 0.88 g/dL (Cohen’s d = 0.88; 95% CI: 0.63, 1.13; I^2^ = 96.17%) in hemoglobin levels among women who received nutrition education. A meta-regression analysis revealed that 61.85% (adjusted R^2^ = 61.85%) of heterogeneity between standardized mean differences was explained by anemia status, intervention duration, and geographic region. Also, pregnant women in the nutrition education group had a 34% (relative risk = 0.66; 95% CI: 0.54, 0.80, I^2^ = 86.85%) lower risk of anemia compared with the control group.

**Conclusion:**

Nutrition education during pregnancy improves compliance with IFAS, increases hemoglobin levels, and reduces the risk of anemia. Therefore, it is crucial to enhance the existing nutrition education program to prevent and control anemia during pregnancy.

**Systematic Review Registration:**

PROSPERO registration no. CRD42023454241.

## INTRODUCTION

Anemia is a low red blood cell count that can cause decreased oxygen delivery to tissues,^1^^,^^2^ which is diagnosed when the hemoglobin concentration falls below 11 g/dL in pregnant women.^2^ In 2021, sub-Saharan Africa and South Asia had the highest prevalence of anemia among women of reproductive ages (western sub-Saharan Africa [47.4%], South Asia [35.7%], and central sub-Saharan Africa [35.7%]).^3^ In 2019, 36.5% of pregnant women were anemic globally.^4^ The severity of the problem was higher in low- and middle-income countries (LMICs).^1^^,^^3–5^

Even if anemia has various causes, it is commonly caused by inadequate iron-rich food intake,^6^ followed by a high frequency of infections,^7–9^ and insufficient intake of vitamins (eg, folate, vitamin B_12_, vitamin A).^10^ Other causes of anemia include genetic disorders (thalassemia and sickle cell anemia), certain medications, and immunologic and noninfectious diseases such as cancer.^11–13^

Anemia during pregnancy leads to maternal complications such as inadequate weight gain, preterm labor, placental issues, cardiac problems, hemorrhage, infection risk, and reduced physical capacity. Also, anemia in the mother increases health risks for newborns, such as premature birth, low birth weight, growth restriction, and anemia.^14–17^

To prevent the consequences of anemia, the World Health Organization (WHO) recommends implementing daily or intermittent oral iron–folic acid (IFA) supplementation (IFAS)^18^ and promoting essential nutrition actions^19^ with other multisectoral collaboration for nutrition-sensitive and specific interventions including nutrition education.^20^ To meet United Nations (UN) Sustainable Development Goals (SDGs)^21^ and global nutrition targets,^22^ this type of comprehensive approach is highly appropriate in LMICs. In response, governments and stakeholders in LMICs have implemented prevention and control approaches, including increasing the intake of iron-rich foods, fortifying food, providing micronutrient supplementation (iron, folic acid, and others), and strengthening disease prevention,^5^^,^^23^ such as nutrition education and counseling during antenatal care, which are linked to SDGs.^21^

Previous research and reviews revealed that the intake of IFA supplements and/or other public health and nutrition-specific interventions (eg, deworming, treating malaria, and vitamin A supplementation) reduces the risk of anemia among pregnant women.^24–27^ A systematic review showed that nutrition education has a positive influence on maternal, neonatal, and child health outcomes during pregnancy.^28^ This review underscored the efficacy of nutrition education and/or counseling in reducing the prevalence of anemia among pregnant women, drawing from a limited number of studies. However, it did not thoroughly examine its impact on compliance with IFAS or changes in hemoglobin levels.

Implementing both nutrition education and/or IFAS or micronutrient supplementation plays a role in preventing anemia. However, stakeholders prioritized nutrition-specific interventions to achieve SDGs, with limited attention to and promotion of health and nutrition education components.^29^ The use of nutrition education along with IFAS intervention not only tackles immediate consequences but also breaks the cycle of anemia across generations.^18^^,^^28^^,^^30^^,^^31^

As a prevention strategy, nutrition education plays a crucial role in increasing knowledge about and practice related to anemia prevention and control. Nutrition education may help reduce forgetfulness regarding IFA tablet intake and increase awareness of the minor and temporary side effects of IFA tablets, leading to higher IFA tablet intake among pregnant women. This, in turn, leads to elevated hemoglobin levels, and ultimately helps to prevent and control anemia during pregnancy and later stages of life.^32–35^

Apart from individual small-scale studies, there is a lack of a comprehensive systematic review or meta-analysis examining the effect of nutrition education on compliance with IFAS, changes in hemoglobin levels, or the prevalence of anemia in LMICs. In filling this research gap, the authors performed a systematic review and meta-analysis, which aimed to generate evidence from relevant research articles to show the impact of nutrition education during pregnancy on IFAS compliance, hemoglobin change, and the prevalence of anemia in LMICs.

## METHODS

### Design and Protocol Development

The systematic review and meta-analysis was conducted and reported according to the Preferred Reporting Items for Systematic Reviews and Meta-Analyses (PRISMA) statement (Table S1).^36^ The review protocol was registered in the International Prospective Register of Systematic Reviews database (PROSPERO no. CRD42023454241).^37^

### Eligibility Criteria

Studies that reported the effect of nutrition education during pregnancy on IFAS compliance rates, change in hemoglobin/hematocrit, and/or prevalence of anemia were considered. This review encompasses all randomized controlled trials (RCTs) and quasi-experimental studies (including pre-post comparisons and a control group). Only peer-reviewed published articles were included in this review. The study settings were limited to LMICs, as per the World Bank definition.

This review excluded studies on nonpregnant participants, those not published in English, or those conducted in developed countries. Also, studies that determined the magnitude of IFAS compliance, mean hemoglobin, and/or prevalence of anemia without a nutrition education intervention were excluded from this review. In addition to these, studies that used IFAS or any other micronutrient supplementation as an intervention were excluded from this review.

### Study Source and Search Strategies

Potential research articles were identified through systematic searches using electronic bibliographic databases for published work on Embase, Ovid Medline, Scopus, Web of Science, Health and Medical Collection (ProQuest), and Google Scholar. Additional manual searches were also conducted through reference lists of the candidate studies. The search was limited to the English language and publication year 2000 and later.

The search terms were grouped into 4 major categories: population, intervention, comparator, outcome, and study setting (PICOS) using the following search terms: “pregnant women” AND “nutrition education” AND “anaemia OR “haemoglobin level” OR “IFAS compliance”” AND “Developing country,” as shown in Table 1. The detailed search strategy for each selected database is shown in Table S2. While conducting a Google Scholar search, specific key words or phrases, along with the “intitle” modifier, such as “IFAS compliance,” “anaemia,” or “haemoglobin,” were used. Additionally, further searches were conducted to identify related articles from the selected articles on Google Scholar. The initial searches were conducted on September 11, 2023, with the final update searches performed on November 16, 2023.

**Table 1.** PICOS Criteria for Inclusion of Studies

Parameter	Criterion
Population	Pregnant women
Intervention	Nutrition education
Comparator	Not involved in nutrition education program
Outcomes	Iron–folic acid supplementation (IFAS) compliance, mean hemoglobin level, and/or anemia
Study settings	Low- and middle-income countries list

### Study Selection and Data Collection

All potential studies were initially retrieved from electronic databases and subsequently imported into Covidence for data management and screening. Covidence was used to exclude duplicate studies in the initial phase. Subsequently, 2 authors (M.T.E. and G.F.) independently conducted screening, progressing from title and abstract to full-text screening according to the eligibility criteria. Any disagreements or conflicts between reviewers during this process were resolved by a third author (P.M.).

### Data Extraction and Management

Data extraction was conducted using Microsoft Excel (Microsoft Corporation, Redmond, WA, USA) by 2 reviewers (M.T.E. and G.F.) independently. In case of differences, a third reviewer (P.M.) was involved in the final decision. The data-extraction tool for experimental studies was adapted from the Cochrane Handbook for systematic reviews of interventions.^38^ For each study, the authors extracted year of publication, study design, sample size, the prevalence of anemia, hemoglobin/hematocrit level, IFAS compliance rate, study location (country), intervention type, and participant characteristics, including sociodemographics.

### Outcome Variables

This review primarily determined the overall effect of nutrition education on IFAS, change in hemoglobin, and/or prevalence of anemia. Anemia status was determined if the pregnant woman’s hemoglobin level was less than 11.0 g/dL. The compliance rate was determined by counting the number of days a pregnant woman used IFAS throughout her pregnancy.

### Type and Components of the Nutrition Education

This review focuses on nutrition education as the intervention aimed at informing pregnant women about anemia prevention and control activities during pregnancy. The provided nutrition education includes regular oral information on the causes, signs, symptoms, and consequences of anemia, along with strategies for the prevention and control of anemia across all of the included studies. These strategies emphasize the importance of dietary changes during pregnancy, such as increasing the variety and frequency of meals, boosting consumption of iron-rich foods, deworming, and advocating the use of IFA supplements.

Nutrition education typically involves direct (face-to-face) or indirect (eg, phone calls and video conferencing) teaching methods through repeated oral sessions with and without visual aids such as pictures, pamphlets, videos, etc, for certain periods. These sessions are conducted in clinical and community settings by investigators or healthcare professionals who are trained for this purpose. Studies relying solely on pamphlets, visual aids, media outlets, promotions, or one-time interventions were excluded from this review.

### Assessment of the Quality of the Individual Studies

The methodological quality of the included experimental studies was assessed using the revised Joanna Briggs Institute (JBI) critical appraisal or quality-assessment tools. The JBI critical appraisal tool has 13 and 9 questions for critical appraisal of RCTs^39^ and quasi-experimental studies, respectively.^40^ The quality of the included studies was assessed by 2 reviewers (M.T.E. and G.F.) and any disagreement and problems in the process of appraisal and article rating were resolved by a third reviewer (P.M.).

### Statistical Analysis

After extraction, the data were imported to Stata/MP version 17.0 software (StataCorp LLC, College Station, TX, USA) for further descriptive and meta-analysis. The pooled estimates of the standardized mean difference (SMD) of hemoglobin, IFAS compliance (odds ratio [OR]), and prevalence of anemia (risk ratio) were assessed using a random-effects model. Heterogeneity between studies due to study quality, sample size, method, and different outcome measurements is common but needs to be addressed during meta-analysis.^41^^,^^42^ Hence, the presence of heterogeneity/statistical significance was assessed using *I*^2^ statistics. The levels of heterogeneity were categorized as low, medium, and high using corresponding *I*^2^ values of 25%, 50%, and 75%, respectively. A random-effects model was chosen instead of a fixed-effects model due to the high level of heterogeneity,^43^ study variability, and generalizability. During the meta-analysis of continuous outcome variables, standardizing the mean difference was used to address variations in the units of measurement across the included studies.^44^

To enhance consistency in estimating aggregated effect sizes from primary studies, a subgroup analysis was performed, considering intervention timing, duration, participant type (anemic and nonanemic participants), and the person who delivers the nutrition education. Random-effects models using the Restricted Maximum Likelihood (REML) method, the default approach, were used to pool the effects of nutrition education on the outcome variables due to high heterogeneity,^43^ study variability (true effect sizes vary from study to study due to differences in study populations, interventions, methodologies, etc), and to make the result more generalizable. Also, univariate and multivariable meta-regression was used, incorporating factors such as publication year, sample size, participant type, and WHO region. Publication bias was assessed through funnel plot examination, sensitivity test, and Egger’s statistical test (*P* <.05).^45^ When Egger’s test was statistically significant, the result was further explored using nonparametric trim-and-fill analysis for random-effects models.^46^

## RESULTS

### Study Selection and Identification

**Figure 1.**
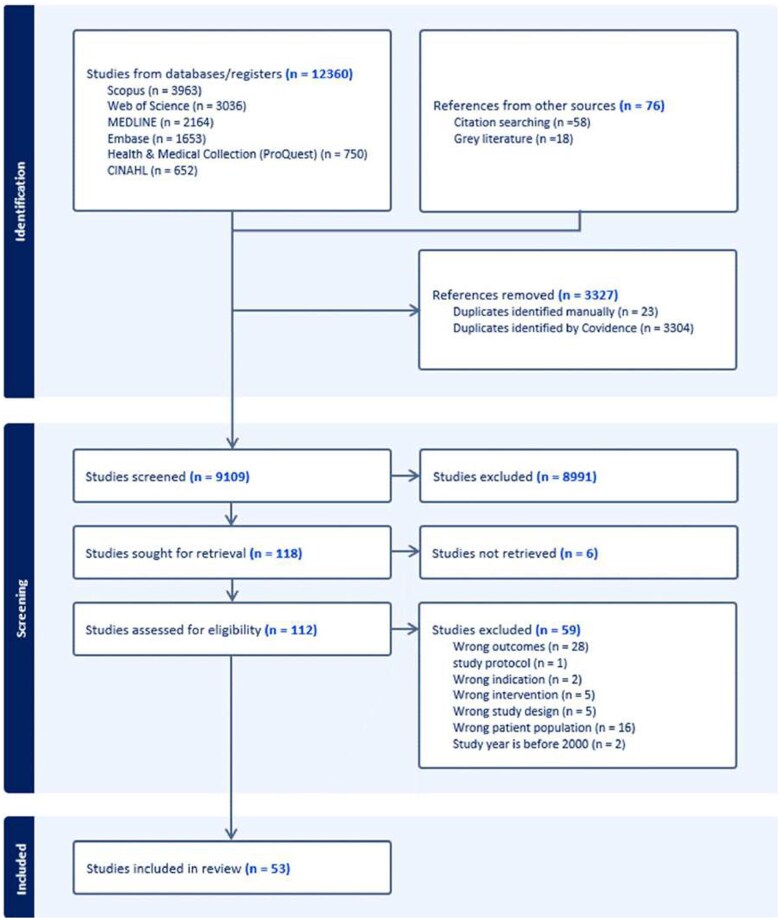
PRISMA (Preferred Reporting Items for Systematic Reviews and Meta-Analyses) Flow Diagram of Article Selection for the Systematic Review and Meta-analysis

From all of the searched databases, 12 436 articles (12 360 and 76 references from databases and other sources, respectively) were imported into Covidence for screening. From the total, 3327 duplicates were removed through Covidence and manual checks. Title and abstract screening led to the exclusion of 8991 studies. During the full-text screening, 65 articles were excluded due to incorrect study design and lack of relevant information. Finally, 53 peer-reviewed articles were included for qualitative and quantitative synthesis (Figure 1).

### Characteristics of Included Studies

The included studies were published from 2000 to 2023, with the majority (64.15%) published in 2018. India contributed to the most included studies (*n* = 12, 22.64%), followed by Indonesia (*n* = 11, 20.75%) and Egypt (*n* = 11, 20.75%). Health facility–based studies accounted for 77.36% (*n* = 41). Of these, 23 studies utilized an RCT study design,^47–69^ while 21 studies used quasi-experimental designs with controls.^35^^,^^70–89^ The remaining 9 studies were quasi-experimental designs with a pre-post design.^90–98^ From 53 studies, 14 (26.41%) had interventions lasting fewer than 12 weeks.^47^^,^^50^^,^^62^^,^^64^^,^^70^^,^^73^^,^^75–77^^,^^80^^,^^87^^,^^88^^,^^91^^,^^95^

Overall, this review included a total of 13 475 pregnant women participants. The smallest and the largest sample sizes across the studies were 28 and 2000, respectively.^69^^,^^73^ Out of 53 studies, 14 presented the IFAS compliance rates, 39 reported the mean hemoglobin levels along with SDs, and 21 provided information on the prevalence of anemia. It is crucial to note that a single study may address 1 or more of these findings. Most of the studies (36 [67.92%]) were conducted in Asian countries (Table 2). However, 4 studies out of the 53^50^^,^^62^^,^^97^^,^^98^ were excluded from further analysis due to zero frequency in either arm of the study outcomes (IFAS compliance or prevalence of anemia) or not reporting the mean ± SD of the hemoglobin level.

**Table 2.** Characteristics of the Included Studies in LMICs, 2023

Study no.	Study (publication year)	Study location	Sample size, *n*	Study setting	Study design	Intervention duration, wk	Study quality score
1.	Wakwoya et al (2023)^65^	Ethiopia	326	Health facility	RCT	24	High
2.	Koné et al (2023)^57^	Côte d’Ivoire	473	Health facility	RCT	16	High
3.	Arifah et al (2023)^50^	Indonesia	44	Health facility	RCT	7	High
4.	Ramachandran et al (2023)^76^	India	117	Health facility	Quasi-experimental with control	4	High
5.	Sharma et al (2023)^62^	India	250	Health facility	RCT	4	High
6.	Shetty et al (2023)^63^	India	139	Health facility	RCT	12	High
7.	Soifah et al (2022)^75^	Thailand	50	Health facility	Quasi-experimental with control	6	High
8.	Abd Rahman et al (2022)^60^^,^^a^	Egypt	300	Health facility	Quasi-experimental pre-post	12	Medium
9.	Ahmad et al (2022)^49^	Indonesia	110	Health facility	RCT	12	Medium
10.	Ariyani et al (2022)^51^	Indonesia	145	Health facility	RCT	12	Medium
11.	Elsharkawy et al (2022)^53^	Saudi Arabia	196	Health facility	RCT	12	High
12.	Abd Rahman et al (2022)^60^	Malaysia	94	Health facility	RCT	12	High
13.	Sontakke et al (2022)^68^	India	240	Health facility	RCT	12	high
14.	Mohamed Elsayed Ahmed et al (2021)^93^	Egypt	100	Health facility	Quasi-experimental pre-post	12	medium
15.	El-Kholy et al (2021)^96^^,^^a^	Egypt	60	Health facility	Quasi-experimental pre-post	12	medium
16.	Ilboudo et al (2021)^55^	Burkina Faso	553	Community	RCT	16	High
17.	Nguyen et al (1) (2021)^58^	India	1849	Community	RCT	18	High
18.	Adje et al (2020)^87^	Nigeria	275	Health facility	Quasi-experimental with control	8	High
19.	Brawy et al (2020)^70^	Egypt	180	Health facility	Quasi-experimental with control	8	High
20.	Hassan et al (2020)^72^	Malaysia	162	Health facility	Quasi-experimental with control	12	High
21.	Husna et al (2020)^54^	Indonesia	40	Health facility	RCT	16	Medium
22.	Nadziroh et al (2020)^73^	Indonesia	28	Health facility	Quasi-experimental with control	4	High
23.	Nahrisah et al (2020)^35^	Indonesia	140	Community	Quasi-experimental with control	15	High
24.	Sundayani et al (2020)^91^	Indonesia	30	Health facility	Quasi-experimental pre-post	2	Medium
25.	Singh et al (2020)^67^	Nepal	413	Community	RCT	12	High
26.	Yani et al (2020)^90^	Indonesia	51	Community	Quasi-experimental pre-post	12	Medium
27.	Abdel-Ati et al (2019)^83^	Egypt	120	Health facility	Quasi-experimental with control	16	High
28.	Dawood and Ali (2019)^71^	Iraqi	60	Health facility	Quasi-experimental with control	12	High
29.	Abd Elhaleem Ebraheem Elagamy et al (2019)^95^^,^^a^	Egypt	210	Health facility	Quasi-experimental pre-post	8	Medium
30.	Ouedraogo et al (2019)^92^	Niger	555	Community	Quasi-experimental pre-post	24	High
31.	Sunuwar et al (2019)^80^	Nepal	107	Health facility	Quasi-experimental with control	10	High
32.	Abujilban et al (2018)^47^	Jordan	200	Health facility	RCT	4	High
33.	Esmat et al (2018)^94^	Egypt	48	Health facility	Quasi-experimental pre-post	12	Medium
34.	Shafagat et al (2018)^61^	Iran	120	Health facility	RCT	12	Medium
35.	Abdel-Mageed et al (2017)^84^^,^^b^	Egypt	180	Health facility	Quasi-experimental with control	12	High
36.	Heryadi et al (2017)^88^^,^^b^	Indonesia	192	Health facility	Quasi-experimental with control	4	High
37.	Nguyen et al (2) (2017)^69^	Bangladesh	2000	Community	RCT	28	High
38.	Deshmukh and Patange (2016)^52^	India	90	Community	RCT	16	Low
39.	Shivalli et al (2015)^79^	India	86	Community	Quasi-experimental with control	12	High
40.	Widyawati et al (2015)^66^	Indonesia	354	Health facility	RCT	24	Medium
41.	Khorshid et al (2014)^56^	Iran	116	Health facility	RCT	12	Medium
42.	Saifuddin et al (2014)^77^	Indonesia	60	Health facility	Quasi-experimental with control	8	High
43.	Pai et al (2013)^59^	India	79	Health facility	RCT	12	High
44.	Abd El Hameed et al (2012)^97^^,^^a^	Egypt	200	Health facility	Quasi-experimental pre-post	12	Medium
45.	Noronha et al (2012)^74^	India	129	Health facility	Quasi-experimental with control	12	High
46.	Senanayake et al (2010)^78^	Sri Lanka	218	Health facility	Quasi-experimental with control	14	High
47.	Susheela et al (2010)^64^	India	205	Health facility	RCT	2	Medium
48.	Ndiaye et al (2009)^89^	Senegal	371	Community	Quasi-experimental with control	12	High
49.	Adhikari et al (2008)^48^	Nepal	284	Health facility	RCT	12	Medium
50.	Garg and Sushma (2006)^86^^,^^b^	India	96	Community	Quasi-experimental with control	16	High
51.	Gadallah et al (2002)^85^	Egypt	100	Health facility	Quasi-experimental with control	12	High
52.	Al-Tell et al (2001)^82^	Egypt	102	Health facility	Quasi-experimental with control	20	High
53.	Abel et al (2000)^81^	India	828	Community	Quasi-experimental with control	52	High

a Did not use during prevalence of anemia meta-analysis.

b Not used during IFAS compliance rate meta-analysis.

Abbreviations: IFAS, iron–folic acid supplementation; LMIC, low- and middle-income country; RCT, randomized controlled trial.

### Quality of the Study

The quality of the studies was assessed using the JBI critical appraisal tool for RCTs and quasi-experimental studies. In this review, the majority of the studies (*n* = 36, 67.92%) had good quality,^35^^,^^47^^,^^50^^,^^53^^,^^55^^,^^57–60^^,^^62^^,^^63^^,^^65^^,^^67–89^^,^^92^ 16 studies (30.19%) had medium quality,^48^^,^^49^^,^^51^^,^^54^^,^^56^^,^^61^^,^^64^^,^^66^^,^^90^^,^^91^^,^^93–98^ and only 1 study (1.89%) had low quality^52^ (see Table 2). During the analysis, none of the studies were excluded based on methodological quality (Table S3).

### Meta-analysis Results

#### Pooled Effect of Nutrition Education on the IFAS Compliance Rate

**Figure 2.**
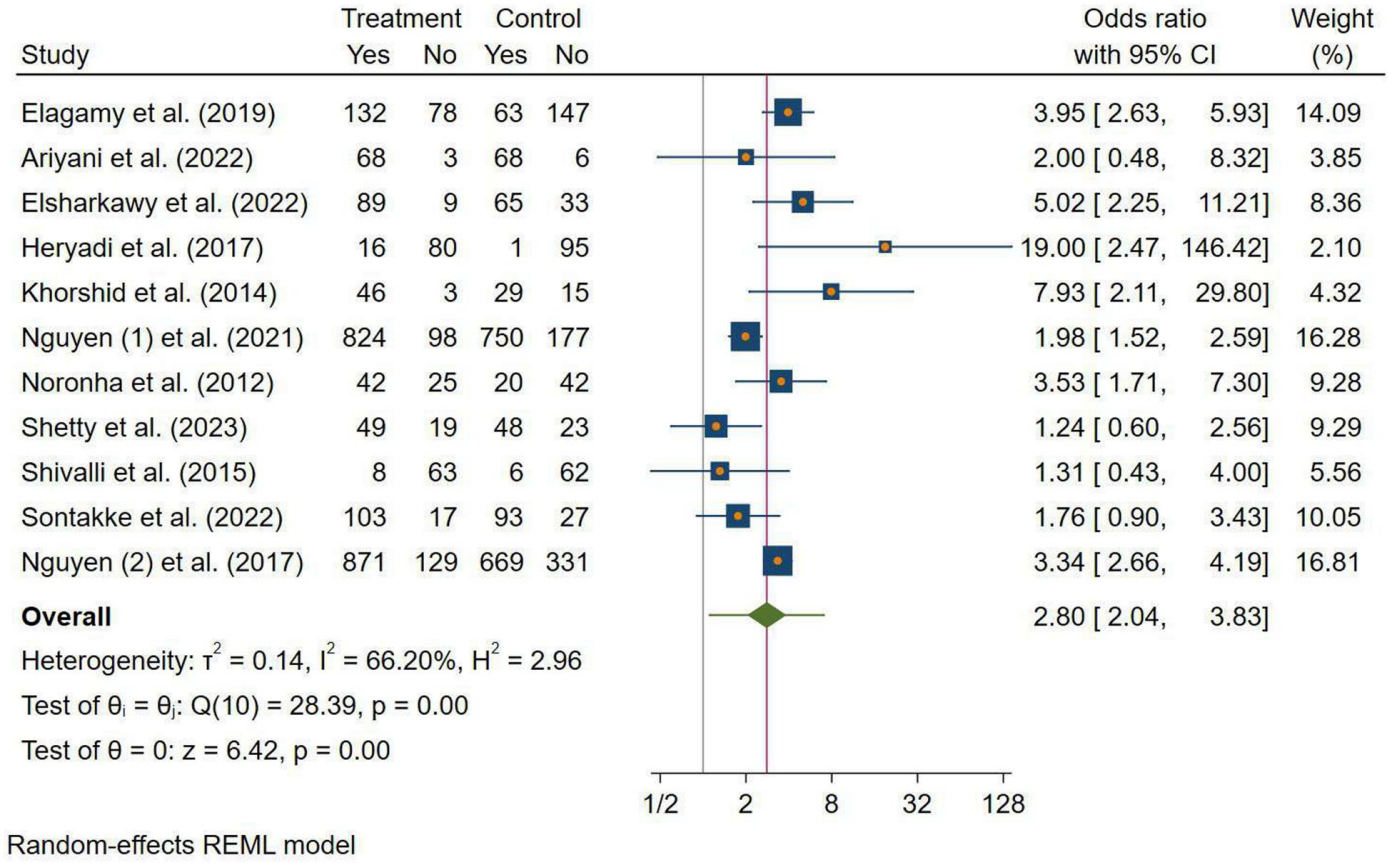
Forest Plot Illustrating the Impact of Nutrition Education During Pregnancy on IFAS Compliance Rate in LMICs, 2023. Abbreviations: IFAS, iron–folic acid supplementation; LMIC, low- and middle-income country; REML, Restricted Maximum Likelihood

The results from 13 studies were initially pooled to assess the impact of nutrition education during pregnancy on IFAS compliance; however, 2 studies^84^^,^^86^ were excluded from further meta-analysis due to zero events in either arm of the study group outcome. As a result, the pooled effect of nutrition education was estimated from 11 studies.^51^^,^^53^^,^^56^^,^^58^^,^^63^^,^^68^^,^^69^^,^^74^^,^^79^^,^^88^^,^^95^ The aggregated result revealed that those who received nutrition education during pregnancy were 2.80 times more compliant to IFAS (OR = 2.80; 95% CI: 2.04, 3.83; *P* < .001) than those who did not. However, moderate heterogeneity between studies was observed (τ^2^ = 0.14, *I*^2^ = 66.20%) (Figure 2).

To evaluate this moderate heterogeneity (*I*^2^ = 66.20%), an in-depth analysis was performed using subgroup analysis. Subgroup analysis showed that the effect of nutrition education on IFAS compliance during pregnancy did not significantly differ based on study participants, study design, study setting, or study quality. During subgroup analysis, the OR varied from 2.37 to 3.98, a range within the 95% CI between 1.03 and 12.07. However, studies conducted in health facilities, moderate-quality studies, and those using direct and indirect nutrition education had an *I*^2^ < 60%. The highest pooled OR was observed among studies with moderate quality (OR = 3.98; 95% CI: 2.74, 5.80), while the lowest was found among studies conducted in community settings (OR = 2.37; 95% CI: 1.48, 3.79) (see Table 3).

**Table 3.** Subgroup Analysis of SMD Hemoglobin, IFAS Compliance, and Prevalence of Anemia in LMICs, 2023

Variables	**Study characteristics/participants**	Included studies	Effect size (95% CI)	Heterogeneity, *I*^2^, *P* value
Subgroup pooled rate of IFAS compliance (*n* = 11, OR)				
Study design	RCT	7	2.54 (1.72, 3.76)	71.73%, <.001
	Quasi-experimental with control	3	3.53 (1.03, 12.07)	68.99%, .07
	Quasi experimental pre-post	1	3.95 (2.63, 5.93)	NA
Study participants	Anemic pregnant women	5	2.76 (1.68, 4.55)	66.54%, .02
	All pregnant women	6	2.84 (1.78, 4.51)	69.65%, .01
Study setting	Health facility	8	3.19 (2.00, 5.07)	59.60%, .02
	Community	3	2.37 (1.48, 3.79)	79.74%, .01
A person who delivers the NE	Trained HCWs	5	2.68 (1.86, 3.86)	70.94%, .01
	Researchers	6	3.33 (1.72, 6.42)	68.33%, .01
Mode of NE delivery	Directly	5	2.74 (1.85, 4.04)	65.54%, .01
	Indirectly	4	2.63 (1.30, 5.35)	75.60%, .01
	Both	2	3.86 (1.71, 8.72)	17.67%, .01
Quality of the study	High	8	2.52 (1.77, 3.60)	68.11%, <.001
	Moderate	3	3.98 (2.74, 5.80)	0.00%, .38
Subgroup pooled mean difference of hemoglobin (*n* = 39, SMD)				
Study design	RCT	15	0.72 (0.34, 1.10)	95.51%, <.001
	Quasi-experimental with control	18	0.96 (0.60, 1.33)	94.72%, <.001
	Quasi-experimental pre-post	6	1.03 (0.29, 1.77)	97.05%, <.001
Study participants	Anemic pregnant women	16	1.28 (0.92, 1.65)	94.56%, <.001
	All pregnant women	23	0.43 (0.24, 0.63)	90.25%, <.001
Study setting	Health facility	30	0.92 (0.65, 1.20)	94.32%, <.001
	Community	9	0.73 (0.15, 1.31)	98.15%, <.001
A person who delivers the NE	Trained HCWs	13	0.86 (0.37, 1.36)	98.16%, <.001
	Researchers	26	0.89 (0.61, 1.17)	93.33%, <.001
Mode of NE delivery	Directly	29	0.91 (0.64, 1.19)	96.05%, <.001
	Indirectly	8	0.60 (0.13, 1.08)	92.55%, <.001
	Both	2	1.45 (−0.83, 3.72)	99.07%, <.001
WHO regions of the countries	South Asia	13	0.62 (0.46, 0.78)	69.73%, <.001
	East Africa	8	1.49 (1.20, 2.13)	94.55%, <.001
	East Asia	4	0.94 (−0.20, 2.08)	97.70%, <.001
	West Africa	3	0.03 (−0.49, 0.54)	96.17%, <.001
	Southeast Asia	11	0.95 (0.43, 1.48)	93.38%, <.001
Study quality	High	28	0.88 (0.56, 1.19)	97.11%, <.001
	Moderate	10	0.90 (0.47, 1.33)	91.51%, <.001
	Low	1	0.79 (0.34, 1.25)	NA
Subgroup analysis on the pooled effects of anemia prevalence (*n* = 12, RR)				
Study design	RCT	4	0.63 (0.41, 0.96)	81.94%, <.001
	Quasi-experimental with control	7	0.63 (0.48, 0.82)	83.87%, <.001
	Quasi experimental pre-post	1	0.88 (0.81, 0.96)	NA
Study setting	Health facility	6	0.55 (0.36, 0.84)	77.30%, <.001
	Community	6	0.73 (0.62, 0.86)	80.64%, <.001
WHO geographic region	South Asia	5	0.55 (0.39, 0.79)	87.17%, <.001
	Northeast Africa	1	0.83 (0.50, 1.40)	NA
	West Africa	5	0.75 (0.60, 0.95)	86.03%, <.001
	Southeast Asia	1	0.25 (0.06, 1.12)	NA
Study quality	High	10	0.68 (0.56, 0.83)	88.34%, <.001
	Moderate	2	0.44 (0.26, 0.75)	0.00%, .43

Abbreviations: HCW, healthcare worker; IFAS, iron–folic acid supplementation; LMIC, low- and middle-income country; NA, not available; NE, nutrition education; OR, odds ratio; RCT, randomized controlled trial; RR = relative risk; SMD = standardized mean difference; WHO, World Health Organization.

A funnel plot and Egger’s regression test were used to identify publication bias. The funnel plot for IFAS compliance (Figure S1a) appeared to be symmetrical, indicating no significant publication bias (*P* = .3638 for Egger’s regression test).

The Galbraith plot indicates a positive impact of nutrition education during pregnancy on the IFAS compliance rate. All studies fell within the 95% CI of the standardized log OR, indicating an absence of potential heterogeneity or outlier studies (Figure S2a). Furthermore, a sensitivity analysis using the “one-leave-out” method demonstrated that excluding any single study did not significantly impact the overall effects of nutrition education on IFAS compliance rates during pregnancy. When each study was omitted individually, the OR varied from 2.65 to 3.01, a range within the CI of the overall OR (OR = 2.80; 95% CI: 2.04, 3.83) (Figure S3a).

#### Pooled Effect of Nutrition Education on Hemoglobin Levels

This meta-analysis contained 39 eligible peer-reviewed articles, which included studies that measured postintervention hemoglobin levels. Of these, 36 studies reported mean hemoglobin levels in mg/dL, 1 study reported the result using g/L,^92^ and 2 studies reported the result using hematocrit levels^75^^,^^87^ with SDs (±SD). Then, the mean difference in hemoglobin levels was standardized using Cohen’s *d* effect size.

**Figure 3.**
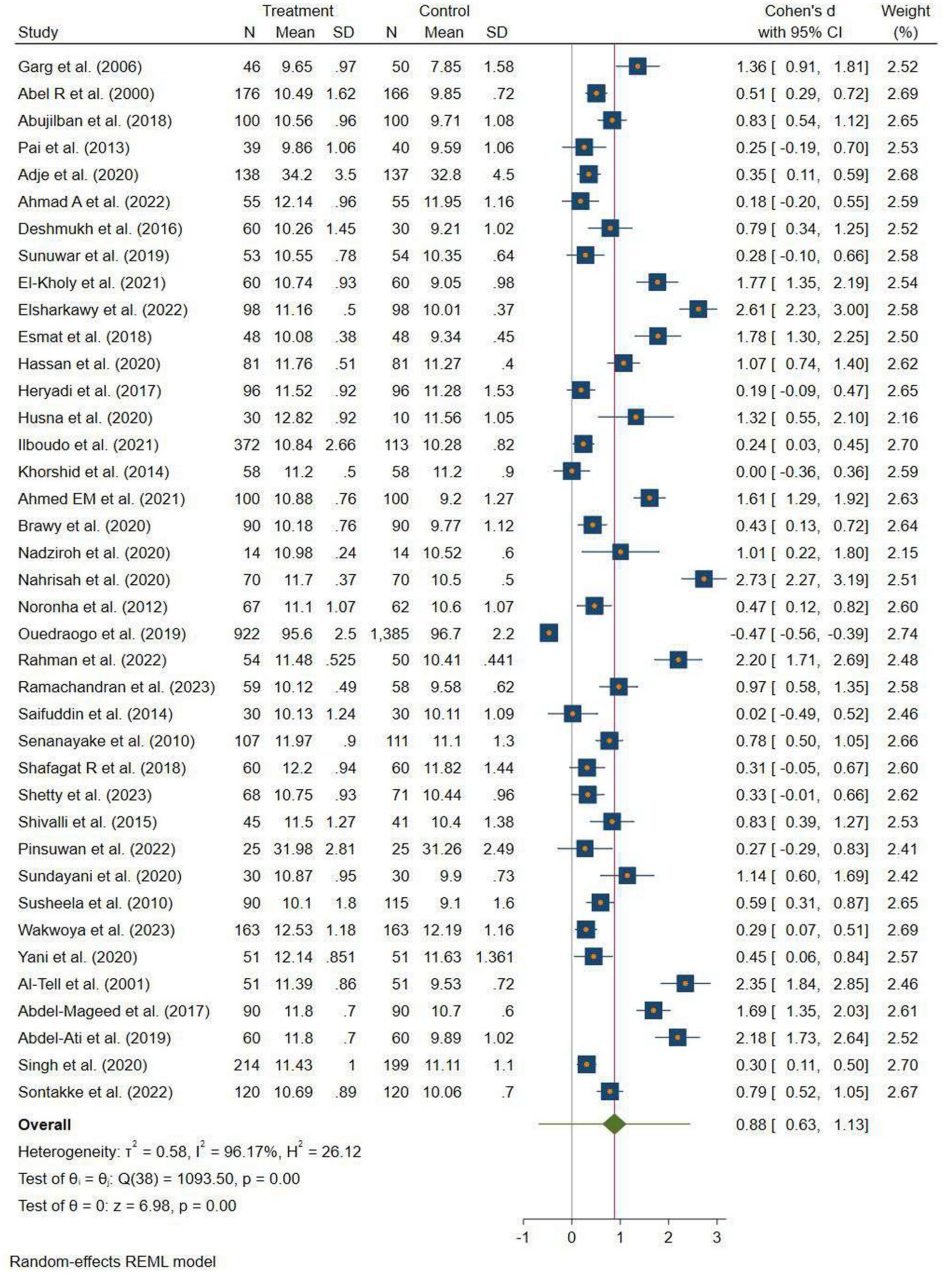
Forest Plot for the Effect of Nutrition Education During Pregnancy on the SMD of Hemoglobin in LMICs, 2023. Abbreviations: LMIC, low- and middle-income country; REML, Restricted Maximum Likelihood; SMD, standardized mean difference

The overall mean (range) hemoglobin level (from 36 studies) between the control and intervention groups was 10.31 g/dL (7.85–12.19 g/dL) and 11.09 g/dL (9.65–12.82 g/dL), respectively. The pooled SDs from 36 studies of the intervention and control groups were 0.93 and 1.05, respectively. The remaining studies reported mean ± SD hemoglobin using g/L (control = 96.7 ± 2.2 g/L vs intervention = 95.6 ± 2.5), and hematocrit level ± SD (control = 32.03 ± 3.50 vs intervention = 33.09 ± 3.16). Following nutrition education, the overall SMD in hemoglobin level in LMICs was 0.88 g/dL (95% CI: 0.63, 1.13 g/dL; *P* < .001) (Figure 3).

The meta-analysis revealed considerable heterogeneity between the studies (τ^2^ = 0.58, *I*^2^ = 96.17%). The findings from the asymmetrical funnel plot (Figure S1b) and Egger’s regression test (*P* < .004) suggested the existence of publication bias. The Galbraith plot shows the positive effect of nutrition education on the SMD of hemoglobin. Of 39 studies, 37 had a standardized Cohen’s *d* of mean hemoglobin level that fell within the 95% CI of the Galbraith plot. The remaining 2 studies^35^^,^^53^ had a standardized Cohen’s *d* outside the 95% CI, which suggests potential outliers or heterogeneity (Figure S2b). Meta-analysis after excluding these outliers did not change the overall heterogeneity between the studies.

On the other hand, the subgroup analysis failed to identify the source of heterogeneity in the estimates of intervention effects on the SMD of hemoglobin (Table 2). Only studies conducted in South Asia showed the lowest (*I*^2^ = 69.73%, *P* < .001) heterogeneity during subgroup analysis of the SMD of hemoglobin. As a result, nonparametric trim-and-fill analysis was used. During trim-and-fill analysis, the estimated impact size from 39 studies remained the same (SMD= 0.88 g/dL) and no studies were imputed.

Univariate and multivariate meta-regression analyses were used for the SMD of hemoglobin to identify potential sources of heterogeneity among the studies. In the multivariable meta-regression analysis, the SMD of hemoglobin was significantly associated with study groups, intervention duration, and WHO geographic region, which explained 61.85% of the variation between the studies (adjusted *R*^2^ = 61.85%). The pooled SMD of hemoglobin was higher in studies involving anemic pregnant women (β = 0.63; 95% CI: 0.28, 0.99). Furthermore, studies conducted in the WHO region of northeast Africa showed a higher pooled SMD of hemoglobin (β = 0.76; 95% CI: 0.28, 1.23) compared with South Asian countries. Additionally, interventions with a duration of fewer than 12 weeks were associated with a decrease in the SMD of hemoglobin by 54% (β = −0.54; 95% CI: −0.92, −0.17) (Table S4).

Furthermore, a sensitivity analysis result indicated that none of the studies significantly influenced the overall pooled estimation of the SMD of hemoglobin level. When 1 study at a time was eliminated, the pooled SMD hemoglobin level from the other studies varied between 0.83 to 0.92 g/dL, which is in line with the overall SMD of the hemoglobin level (Cohen’s *d* = 0.88 g/dL; 95% CI: 0.63, 1.13) (Figure S3b).

#### Pooled Effect of Nutrition Education on the Prevalence of Anemia

**Figure 4.**
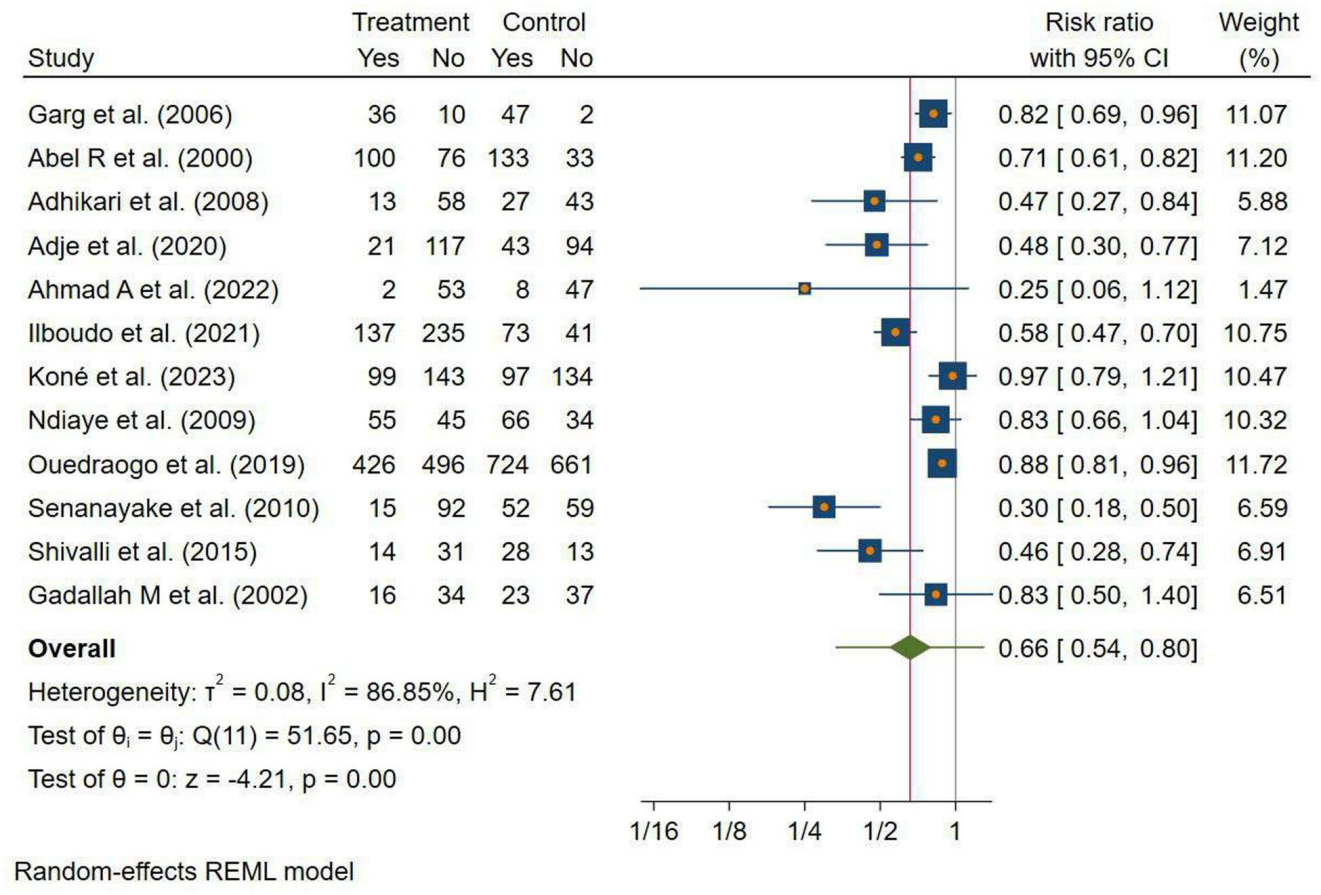
Forest Plot to Illustrate the Effect of Nutrition Education During Pregnancy on the Incidence of Anemia in LMICs, 2023. Abbreviations: LMIC, low- and middle-income country; REML, Restricted Maximum Likelihood

Of the 53 studies, 21 compared the efficacy of nutrition education in preventing anemia in pregnant women. Of 21 studies, 4 studies^95–98^ were excluded from effect size pooling due to the absence of reported events in either study group outcome. Furthermore, 5 studies^64^^,^^66^^,^^71^^,^^74^^,^^76^ were omitted from this analysis due to the inclusion of anemic pregnant women as participants, which makes it difficult to accurately assess the impact of nutrition education on anemia prevalence in individuals already affected by anemia. Consequently, the review’s results from the remaining 12 studies^48^^,^^49^^,^^55^^,^^57^^,^^78^^,^^79^^,^^81^^,^^85–87^^,^^89^^,^^92^ suggested that those who received nutrition education were 34% less likely to have anemia during pregnancy (risk ratio [RR] = 0.66; 95% CI: 0.54, 0.80; *P* < .001). However, the aggregated results showed substantial heterogeneity (τ^2^ = 0.08, *I*^2^ = 86.85%) (Figure 4).

As shown in Figure 4, only 13.15% of the variation among the studies was due to chance. The asymmetrical funnel plot and Egger’s regression test suggested the presence of publication bias or a small study effect (*P* = .0015), indicating a potential cause for overall heterogeneity (Figure S1c). The Galbraith plot from 12 studies suggests a favorable influence of nutrition education in preventing anemia during pregnancy. However, 1 study^78^ was identified as an outlier, which potentially may cause this heterogeneity between the studies (Figure S2c). Pooling the overall effect after excluding this study, only a slight change in the overall RR was observed, and it reduced the heterogeneity by 8.81% (RR = 0.71; 95% CI: 0.61, 0.83; *I*^2^ = 78.04%).

After conducting subgroup analysis, studies with the highest quality had the highest heterogeneity (*I*^2^ = 88.34%, *P* < .001). In addition, the subgroup analysis did not identify the source of heterogeneity (see Table 3). Consequently, a univariate meta-regression analysis was conducted to evaluate the effect of nutrition education on anemia prevalence. Study-level variables, such as intervention duration, study location, WHO region, study design, study quality, and intervention mode, did not impact heterogeneity in the analyses.

Moreover, a sensitivity analysis was conducted for each included study to assess its impact on the overall pooled effect on the prevalence of anemia, yet none of the studies had a significant influence on the overall estimation of the RR (Figure S3c). Since the subgroup and meta-regression analysis failed to identify the source of heterogeneity between studies, a trim-and-fill analysis was further used to assess the cause of heterogeneity.

In the trim-and-fill analysis for anemia prevalence, the initial RR from 12 observed studies did not change (RR= 0.66; 95% CI: 0.54, 0.80). However, after imputing 3 hypothetical missing studies, the funnel plot became more symmetrical. Based on the updated analysis that included the imputed studies (in total, 15 studies), the RR was found to be 0.73 (95% CI: 0.58, 0.92) (Figure S4a). In the enhanced counter trim-and-fill funnel plot analysis, it became apparent that 2 of the 3 imputed studies aligned with regions of statistical significance in the log risk ratio, with a corresponding *P* value greater >.1. This observation may contribute to the observed heterogeneity (Figure S4b).

## DISCUSSION

This study represents the first comprehensive review that summarizes the available evidence regarding the impact of nutrition education during pregnancy on anemia prevention. The results from 51 studies revealed that nutrition education during pregnancy had a positive impact on IFAS compliance rate, increasing hemoglobin level and/or reducing the prevalence of anemia.

This review showed that pregnant women who received nutrition education were 2.79 times more likely to comply with IFAS. Nutrition education has the potential to mitigate factors contributing to poor IFAS compliance. Consequently, it may reduce forgetfulness, enhance understanding of tablet side effects, increase awareness about misconceptions, and encourage behavioral changes, fostering regular antenatal care visits.^29^^,^^56^^,^^99–102^ During nutrition education programs/sessions, pregnant women receive vital lessons that play a crucial role in enhancing knowledge, changing behavior, and practicing preventive measures to control anemia during pregnancy.

Furthermore, this systematic review and meta-analysis demonstrates that providing nutrition education during pregnancy elevates hemoglobin levels and lowers the prevalence of anemia among pregnant women by 34% in LMICs. Previous studies have shown that the implementation of nutrition-specific and -sensitive interventions have a positive effect on the prevention and control of anemia during pregnancy.^24^^,^^25^ A review of nutrition education and/or counseling before and during early pregnancy proves effective in mitigating the impact of adverse maternal and newborn outcomes, including anemia.^28^ This review does not cover the effects of nutrition education on compliance with IFAS or changes in hemoglobin levels. This study’s findings aligned with recommendations from the WHO.^18^^,^^19^ The possible reason might be changes in behavioral practices after nutrition education programs, such as increasing dietary diversity^103^ and intake of iron-rich foods and micronutrient supplementation including IFAS.^104^

According to findings from this systematic review and meta-analysis, combining nutrition education with IFAS proves to be effective in the prevention and control of anemia during pregnancy. On the other hand, low IFAS compliance^105^^,^^106^ and high prevalence of anemia^4^^,^^22^ during pregnancy remain a significant public health challenge in LMICs. The combination of nutrition education with other programs makes it difficult to determine its impact independently. Also, whether either individual counseling or group education have the best effect is not well established.^107^ Enhancing the effectiveness of nutrition education for anemia prevention in LMICs requires addressing healthcare workers’ knowledge and skill gaps; implementing continuous supervision and feedback; increasing women’s awareness of anemia prevention, control, and its consequences; dispelling misconceptions about IFAS; and improving access and quality of antenatal care.^29^^,^^108^ Ongoing comprehensive research on the quality of nutrition education, assessment, and monitoring is crucial for the effectiveness of these measures.

Finally, the heterogeneity of the studies was significant when pooling the effect of nutrition education on change in hemoglobin levels and the prevalence of anemia. While heterogeneity is common during meta-analysis,^41^^,^^42^ it might be due to differences in the intervention duration and intensity of the nutrition counseling provided, the different setups for delivering education messages (health intuitions, community, or both), the mode of delivering nutrition education messages, and differences in study geographical areas.

### Strengths and Limitations of the Review

This study has strengths and limitations. It offers valuable insights into the influence of nutrition education during pregnancy on IFAS compliance, hemoglobin levels, and the prevalence of anemia in LMICs using meta-analysis. This is the first comprehensive review that included subgroup analysis, meta-regression, and assessment of publication bias to identify the source of heterogeneity.

Despite its strengths, significant heterogeneity was observed when combining the effects of nutrition education on the SMD of hemoglobin levels and the prevalence of anemia. This heterogeneity challenged the precision of the pooled estimate and remained unidentified by subgroup analysis. However, meta-regression analysis indicated that some of the study-level variables contributed to approximately two-thirds of between-study heterogeneity.

Furthermore, most of the study designs were quasi-experimental with a control group. Additionally, the mode of delivery for nutrition education (orally alone and with visual aids) was carried out by researchers and trained healthcare workers, which could lead to variability in information delivery and consistency. Furthermore, all included studies’ nutrition components followed the same protocol, but there may be variation and inconsistency in the delivery of nutrition education sessions. Even though the outcomes were measured objectively in all studies, these differences might have a confounding impact on the pooled outcomes.

## CONCLUSION

In conclusion, nutrition education programs during pregnancy significantly enhance IFAS compliance, increase hemoglobin levels, and/or reduce the prevalence of anemia in LMICs.

### Implications for Practice

The findings of this review indicate the efficacy of nutrition education interventions during pregnancy in preventing and controlling anemia. Therefore, it is crucial to strengthen the existing nutrition education programs or incorporate them into routine antenatal care if such education is not already being implemented. It is also necessary to closely monitor and evaluate the nutrition education programs to identify implementation-related gaps.

### Future Research

Further research is essential to determine the quality of nutrition education programs and factors that hinder the current nutrition education programs during pregnancy. Future studies should assess gaps in service implementation, quality of nutrition services, quality of nutrition counseling, and healthcare providers’ knowledge and practices.

## Supplementary Material

nuae170_Supplementary_Data

## Data Availability

Data described in the manuscript will be provided based on reasonable request.

## Study Registration and Protocol

This systematic review and meta-analysis protocol has been registered in PROSPERO (2023) as CRD42023454241. The review protocol can be accessed online at https://www.crd.york.ac.uk/prospero/display_record.php?ID=CRD42023454241. After the initial review, the study protocol will be amended.
