# Supplementary material for: Effect of Nutrition Education During Pregnancy on Iron–Folic Acid Supplementation Compliance and Anemia in Low- and Middle-Income Countries: A Systematic Review and Meta-analysis
Source: Nutr Rev. 2024 Nov 14;83(7):e1472–87. doi: 10.1093/nutrit/nuae170 (PMC12166189; doi:10.1093/nutrit/nuae170)
Supplement: nuae170_Supplementary_Data [file nuae170_supplementary_data.zip › nuae170_Supplementary_Data/Figure S1 Funnel plots.pdf]

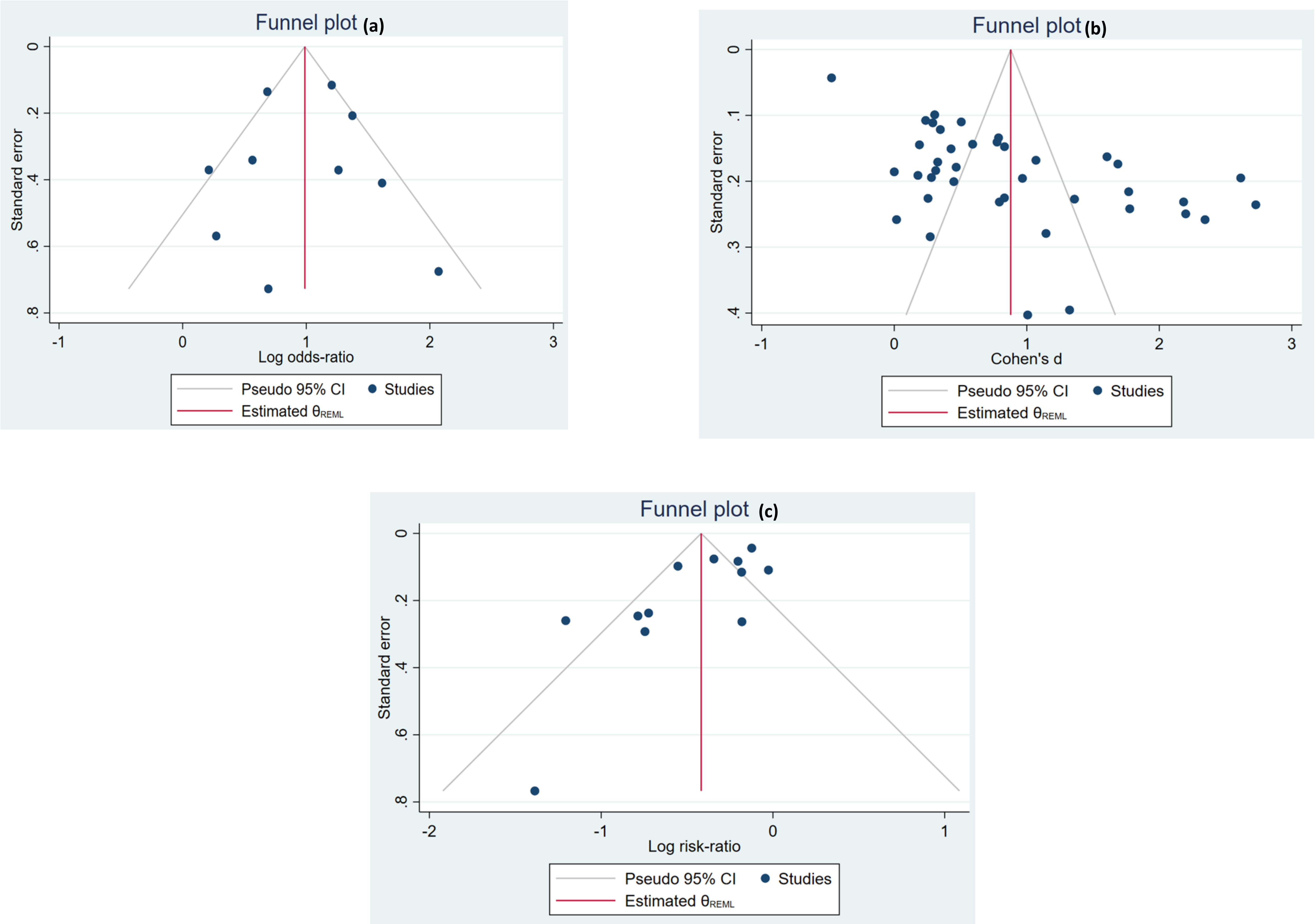

Figure S1: Funnel plot for publication bias on pooled of nutrition education effect on IFAS compliance (**a**), haemoglobin change (**b**), and incidence of anaemia (**c**) in LMICs, 2023.
